# Colposuspension in girls with refractory stress-incontinence, a critical evaluation of a last-resort treatment

**DOI:** 10.3389/fruro.2024.1442599

**Published:** 2024-11-29

**Authors:** Frank-Jan van Geen, Anka Nieuwhof-Leppink, Aart Klijn, Laetitia de Kort, Rafal Chrzan

**Affiliations:** ^1^ Department of Urology, University Medical Center Utrecht, Utrecht, Netherlands; ^2^ Department of Pediatric Urology, Wilhelmina Children’s Hospital, Utrecht, Netherlands; ^3^ Department of Pediatric Urology, Jagiellonian University Medical College, Krakow, Poland

**Keywords:** girls, refractory incontinence, colposuspension, last resort, stress urinary continence

## Abstract

**Introduction:**

We previously presented promising results with a Burch-type colposuspension (BC) in a heterogeneous group of girls with therapy-resistant daytime incontinence (DUI). In view of our clinical observations, we expect that a small group of girls with refractory DUI based on stress-urinary incontinence (SUI) might also benefit from a BC in order to achieve continence.

**Objective:**

To assess the (long-term) effect of BC on refractory DUI in girls with SUI, and to identify predictive factors for success.

**Study design:**

A retrospective chart study including all girls with refractory DUI who underwent an open or laparoscopic BC at our tertiary referral center between 2003-2017 (*n*=34) was performed. Patients were considered refractory after having failed all non-invasive treatment methods. The main outcome measurement was continence, expressed as the percentage of children with decreased incontinence at post-surgical follow-up without any additional treatment (complete response 4-6 months after surgery). Additionally, a cross-sectional follow-up was carried out, assessing the long-term effect of BC on DUI and patient satisfaction by means of standardized questionnaires.

**Results:**

Complete continence after surgery was achieved in 12% (4/34) and 12% (4/34) showed a decrease in frequency of incontinence episodes. Patients with an abnormal flow pattern more often failed complete continence or improved incontinence. After a mean duration of 8 years, 84% (16/19) still experienced DUI. 68% (13/19) of those patients would opt for the BC again.

**Conclusion:**

Although 84% of children still experienced any degree DUI after a mean duration of 8 years after BC, most patients do not regret the decision of surgical treatment. Given the limited benefit and invasiveness of the procedure, however, we discourage to routinely perform BC in children with refractory DUI and SUI. Our results should be taken into account when discussing expectations and chances of success.

## Graphical Abstract

Effect of surgical treatment on stress-urinary incontinence and patient evaluation at long-term follow-up.

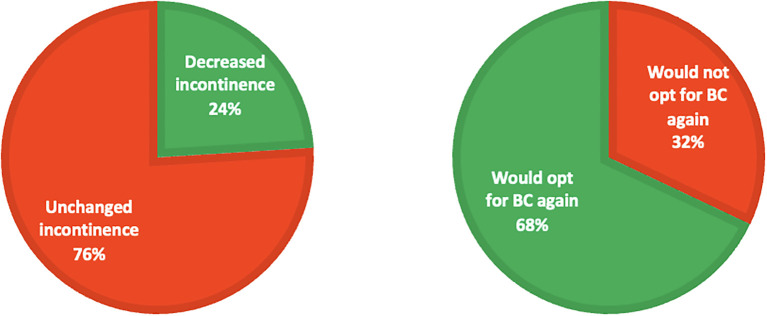

## Introduction

Daytime urinary incontinence (DUI) is a common problem with a major impact on quality of life that affects 7.8% of school aged children (1). Causes of incontinence are various and comprise functional disorders in the vast majority (2, 3). First line treatment is standard urotherapy, a non-surgical, non-pharmacological treatment modality with a success rate of 40% in children (4).

In a small group of girls with DUI however, all non-invasive methods fail. Some of them experience stress-urinary incontinence (SUI), a condition that is rather uncommon in the pediatric population with a prevalence of 8.8% (5). When quality of life is seriously impaired, surgical options which aim to restore the anatomy of the bladder neck, may therefore be considered. Despite no known studies on BC in children with SUI, cure rates in adults with this procedure range from 65 to 90% (6).

Previously, we already presented promising results after Burch-type colposuspension (BC) as last resort option in girls with DUI. Complete continence was achieved in 42%-54% of patients, measured 6 -12 months after the procedure (7–9). Since our former studies included a heterogenic group of patients (e.g. with concomitant complex congenital defects), the primary aim of this study was to evaluate the short and long-term effect of BC on refractory DUI in a larger, more homogenous cohort of girls with SUI. Subsequently, our goal was to identify predictive factors for success such as the degree of incontinence and impact of age.

## Material and methods

The institutional review board committee classified the present study as exempt of the Medical Research Involving Human Subjects Act.

First, data from medical charts has been retrospectively collected and anonymized. All girls with refractory DUI and SUI who underwent an open or laparoscopic BC between 2003-2017 were considered for inclusion, resulting in *n=*34 eligible patients. Excluded were children with neurological lower urinary tract dysfunction, complex anatomical anomalies and/or a history of surgical procedures of the urinary tract. Urodynamic studies were performed before surgery in 97% of patients (33/34). In selected girls, no other cause could be found for the incontinence.

Patients were only considered refractory after having failed standard urotherapy followed by a 10-day in-patient cognitive training program, which is the most intensive form of urotherapy for standard therapy resistant-cases (10, 11). DUI was assessed according to the International Children’s Continence Society (ICCS) standardization paper (12). To detect SUI, an exercise test was performed (7). Children were asked to hop, skip and run up and down stairs with a full bladder, whilst wearing a detector slip to detect involuntary urinary loss during exercise.

There were no definite criteria in terms of frequency or amount of leakage to perform BC. However, all girls were highly motivated and wanted to seize every possible treatment modality to achieve continence. The definitive decision was made in a multidisciplinary setting based on the expert opinion of both the pediatric urologist and the urotherapist in close consultation with the patient and her parents.

Postoperative complications were scored according to the Clavien Dindo classification on a five point scale from I (any deviation from the normal post-operative) to V (death of the patient) (13).

In April 2022, a cross-sectional follow-up was performed. All patients were sent an application form asking for permission to conduct a questionnaire survey by phone. The questionnaires included the International Consultation on Incontinence Questionnaire-Urinary Incontinence (ICIQ-UI) to evaluate the frequency, severity and impact on quality of life of urinary incontinence and the Surgical Satisfaction Questionnaire (SSQ-8) in order to assess patient satisfaction following incontinence surgery.

### Surgical technique

Both the laparoscopic BC, and the open procedure were performed preperitoneally. Under general anesthesia the anterior wall of the vagina, lateral to the urethra, was identified and suspended to Cooper’s ligament with Polyglactin sutures (2–0) as described previously (8, 9).

### Outcome measures

Primary outcome was the percentage of girls with complete continence, defined as no involuntary leakage of urine, or improved incontinence, defined as a decreased frequency of involuntary leakage of urine over the week, without any additional treatment, measured 4-6 months after the procedure.

After 4-6 months postoperatively, a perineal ultrasonography in rest and while straining was performed to assess non-invasively whether the bladder neck was adequately fixed. This was a subjective assessment done by a pediatric urologist as no uniform criteria exist.

The long-term effect of BC was assessed with the ICIQ-UI and DUI was herein defined as any form of involuntary leakage of urine during the day. Patient satisfaction following BC was scored with the SSQ-8 on a 5-point Likert scale; 1=very satisfied, 5= very unsatisfied.

## Results

**Figure 1:**
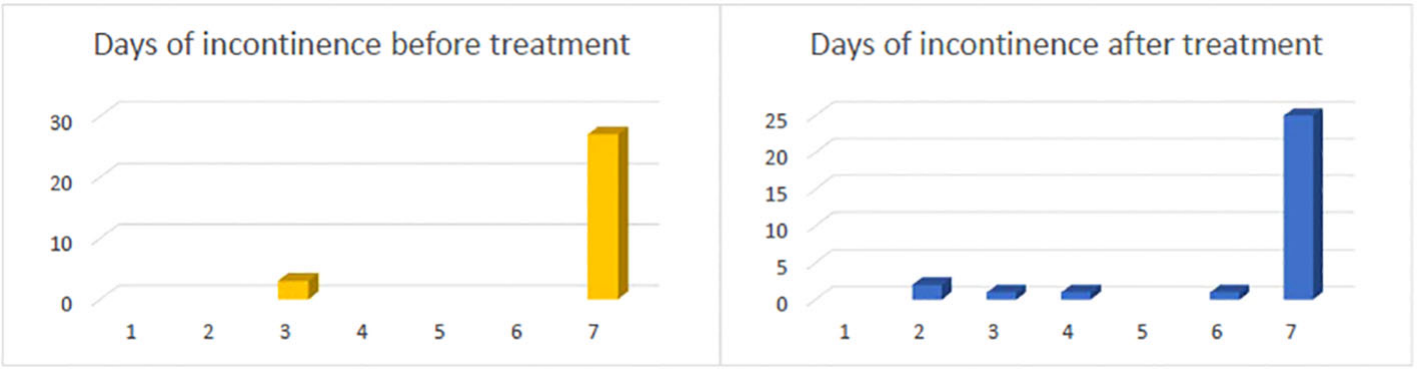
Incontinence frequency per week before and after Burch-type colposuspension *N=30*.

Patient characteristics at baseline are presented in **Table 1**. Complete continence was achieved in 12% (4/34) of all cases. Three of these patients underwent a laparoscopic BC. 12% (4/34) of the children that did not achieve complete continence showed a decrease in frequency of incontinence episodes. However, the majority continued to retain an unchanged degree of incontinence (**Figure 1**).

**Table 1:** Baseline characteristics *N=34*.

	N=34
Mean age at surgery in years (median)	13.4 (14.0)
Laparoscopic procedure	22/34 (65%)
DUI	34/34 (100%)
Drops only	12/28 (43%) 6x N/A
*Uroflowmetry*	33/34 (97%)
Bell-shaped	19/33 (56%) 1x N/A

N/A, Not Applicable.

Postoperative perineal ultrasound was performed in 22 patients; in 72% (16/22) of cases, the bladder neck was judged to be adequately fixed. Within the group of patients who achieved complete continence or improved incontinence 75% (3/4) showed an adequately fixed bladder neck after BC.

Adverse events were reported in 5 of the 34 cases (15%): this involved only grade I complications according to the Clavien-Dindo classification (s.a. post-operative saturation drop without need for treatment and transient neuropathy of the n. femoralis).

Pre-operative patient characteristics by group (complete continence vs improved incontinence vs persistent incontinence) are presented in **Table 2**. Children who achieved complete continence or improved on incontinence (88%) in the majority showed a normal, bell-shaped flow pattern, while patients or reported persistent incontinence after BC (52%) more often showed an abnormal shaped uroflowmetry curve. Patients with minimal urinary loss (drops only) also achieved more often complete continence after BC. Laparoscopic BC lead to improvement in 6/22 (27%) of cases versus in 2/12 (17%) of open procedures.

**Table 2:** Patient stratification on BC outcome in terms of continence *N=34*.

	Complete continence	Improved continence (decrease in frequency of episodes)	Persistent DUI
	n=4	n=4	n=26
Mean age at surgery in years (median)	15.5 (15.0)	11.75 (13.0)	13.31 (14.0)
Laparoscopic procedure	3/4 (75%)	3/4 (75%)	16/26 (62%)
DUI	4/4 (100%)	4/4 (100%)	26/26 (100%)
Drops only	2/2 (100%) 2x N/A	0/3 (0%) 1x N/A	10/23 (43%) 3x N/A
*Uroflowmetry*	3/4 (75%)	4/4 (100%)	25/26 (96%)
Bell-shaped	3/3 (100%) 1x N/A	3/4 (75%)	13/25 (52%) 1x N/A

N/A, Not Applicable.

In total, 19 patients could be reached and participated in the cross-sectional follow-up study. After a mean duration of 8 years, 84% (16/19) still experienced any degree of DUI. The scale of patient satisfaction is presented in **Table 3**, showing that 7/19 (37%) patients were very satisfied or satisfied and 8/19 (42%) were unsatisfied or very unsatisfied. Patients with a higher grade of persistent DUI were more unsatisfied. Despite, 68% (13/19) indicated that they would undergo BC once more if they had to do it all over again.

**Table 3:** Patient satisfaction (mean of 8 years after BC) *N=19*.

How often do you leak urine?	Very satisfied	Satisfied	Neutral	Unsatisfied	Very unsatisfied
Never	2	1	0	0	0
Once a week	0	1	0	2	0
Two-three times a week	0	1	1	0	0
Once a day	0	0	2	0	0
Several times a day	0	2	1	2	3
All the time	0	0	0	0	1
Total	2	5	4	4	4

## Discussion

Our results show that in 12% of all cases complete continence was achieved and 12% of patients showed a decrease in frequency of incontinence, 4-6 months after surgery. Comparison of our data with other studies is difficult due to the heterogenic inclusion criteria and variable sample sizes. Moreover, there is no literature on this type of surgery in the pediatric population. In a previous report of our center, complete continence was reported in 54% after open BC. In this series, also very young girls with complex congenital defects, for example ectopic ureterocele who had BC combined with extensive bladder neck reconstruction, were included (7). In another series of Chrzan et al. continence was achieved in 37.5% of girls with SUI after laparoscopic BC, but only 8 girls were included in this study (9). Dobrowolska-Glazar et al. et al. published a full response rate on incontinence of 42% after BC. However, she measured the outcome 12 months after surgery and including additional treatment (8).

Patients with a preoperative abnormal uroflowmetry curve more often had persistent incontinence. The presence of an abnormal flow pattern may direct towards dysfunctional voiding with secondary detrusor overactivity resulting in predominant urge incontinence instead of pure SUI. Also, dysfunctional voiding with concomitant voiding postponement may have caused overflow incontinence. Understandably from a physiological point of view, suspension of the bladder neck may not be curative in girls with predominant urge incontinence nor in cases with overflow incontinence.

We hoped to demonstrate a difference in effectiveness between a laparoscopic and open approach. However, low success rates and too small sample size prevent clear conclusions. In literature, no significant differences are known in terms of objective cure or patients satisfaction when comparing laparoscopic and open BC (6, 14).

In 22 girls, the postoperative position of the bladder neck was determined by ultrasound in rest and while straining and in 16 the position of the bladder neck was judged as sufficiently fixed. However, there is no literature on the standardization of this measurement, so interpretation of these findings is difficult.

Despite 84% of patients still experiencing any degree of DUI after a mean duration of 8 years, the majority indicated that they would choose BC once more if they had to do it all over again. It seems patients are glad to have explored all possible treatment options to become continent. In our experience, girls who opted for BC, after extensive counseling, were extremely eager to become completely continent, even when the amount of urinary loss was not enormous.

Satisfaction with urological symptoms on the long term showed that slightly more than half of the patients were (very) unsatisfied. The degree of satisfaction correlated with the severity of persistent DUI, illustrating that functional incontinence starting in childhood may have lifelong major impact on quality of life.

Although BC has been described to be effective in adult women with SUI, the results in girls are not convincing. Given the invasive nature of treatment, need for long-term hospitalization with associated costs and limited result on incontinence, the choice for this treatment cannot be justified. We therefore believe that a BC should not routinely be performed in children with refractory DUI and SUI, even with the knowledge that persistent incontinence affects quality of life on the long term. Our results should be taken into account when discussing expectations of additional treatment and chances of success.

### Limitations

The main limitation of this study is the retrospective collection of short time outcomes with a lack of standardized and consistent determination of the amount of urinary loss. Due to this, only complete continence or decrease in incontinence episodes could be identified, instead of (some) improvement on the amount of urinary leakage after operation, hampering a more nuanced description of the outcomes. Furthermore, the limited response for the long-term measurement might have caused a selection bias. It is possible that patients with persistent complaints were more motivated to respond than patients with completely resolved incontinence.

## Conclusion

Although 84% of children still experienced any degree of DUI after a mean duration of 8 years after BC, most patients do not regret the decision of surgical treatment. Given the limited success rate and invasiveness of the treatment, however, we discourage to routinely perform BC in children with refractory DUI and SUI. Our results should be taken into account when discussing expectations and chances of success.

## Data Availability

The data analyzed in this study is subject to the following licenses/restrictions: anonymized only. Requests to access these datasets should be directed to f.vangeen-2@umcutrecht.nl.
